# Mortality from malignant skin melanoma in elderly Brazilians: 2001 to 2016^☆^^☆☆^

**DOI:** 10.1016/j.abd.2020.08.002

**Published:** 2020-11-16

**Authors:** Rodrigo Vasconi Sáez Brown, Danúbia Hillesheim, Yaná Tamara Tomasi, Daniel Holthausen Nunes

**Affiliations:** Universidade Federal de Santa Catarina, Florianópolis, SC, Brazil

**Keywords:** Cutaneous neoplasms, Elderly, Melanoma, Regression analysis

## Abstract

**Background:**

Malignant skin melanoma is a serious public health problem, especially among the elderly population. Knowing the dynamics of the mortality rates of this disease in Brazil is essential to support the creation of public health policies.

**Objective:**

To analyze the temporal trend of mortality from malignant skin melanoma in elderly people in Brazil, from 2001 to 2016.

**Methods:**

This was a descriptive analytical study of mortality rates from malignant skin melanoma in the elderly. The data were obtained from the Mortality Information System, and information related to the population was obtained from the 2010 population census and population estimates from the Brazilian Institute of Geography and Statistics. Mortality coefficients were calculated and simple linear regression analysis of the coefficients was performed by sex and macro-region.

**Results:**

A total 12,712 deaths due to malignant skin melanoma in the elderly were registered. The majority (56.8%) occurred in the male population. In females, a tendency of increase in mortality rates due to malignant skin melanoma was observed in the Northeast (p ≤ 0.001), Midwest (p = 0.002), and Brazil as a whole (p = 0.003). In males, an upward trend was observed in all regions, except for the Southeast region. For both sexes, there was also an upward trend in all regions, with the exception of the Southeast region.

**Study limitations:**

Secondary databases are directly influenced by the quality of death certificate completion and their heterogeneous scope in Brazilian regions.

**Conclusion:**

The increase in mortality indicates a potential public health challenge for the coming decades. The prevention of skin cancer among the elderly should become a priority, mainly through the implementation of preventive measures.

## Introduction

Malignant skin neoplasms are a serious problem and a major challenge for the public health sector. The increase in the incidence and mortality rates of this disease in the last decades is a phenomenon observed in several countries of the world and represents a high impact on the lives of the populations.^1^, ^2^

Among the group of malignant tumors, skin melanoma, a cancer that affects all age groups and has a high potential for severity and dissemination, is noteworthy. In this type of cancer, up to one-fifth of patients develop metastatic disease, highlighting the importance of preventive measures and early diagnosis.^3^ Due to the process of demographic/epidemiological transition and population aging, a significant portion of these cases occur in elderly patients, requiring attention and individualized medical care. The frequent association of other comorbidities, as well as the particularities resulting from senescence, may indicate a greater risk for severe conditions and worse prognosis.^4^, ^5^

In Brazil, cancer is currently the second most frequent cause of death, surpassed only by cardiovascular diseases. In 2018, the National Cancer Institute (Instituto Nacional de Câncer [INCA]) estimated approximately 6,260 new cases of melanoma in the country.^6^ Associated with this growing incidence of malignant cutaneous neoplasms and with an aging population, Brazil faces yet another great challenge: the poor distribution of specialists in dermatology throughout its territory. According to data from the Medical Demography of 2018, there are 8,317 specialists in the country, the vast majority concentrated in the South and Southeast regions (74.5%), with no experience in performing dermatological surgical procedures, and working predominantly in an urban setting and private care.^7^ Due to the vast territorial extension and the remarkable sociodemographic, epidemiological, and cultural diversity, significant differences are noted in the incidence and mortality rates in the different Brazilian macro-regions, thus inferring great complexity in dealing with this disease by the responsible healthcare agents and sectors.^8^

The molecular pathogenesis of malignant melanoma has not yet been fully understood. However, it is known that there is an interface between genetic predisposition, environmental factors, and phenotypic manifestations of the interactions between gene and environment. Among the factors that make the elderly a risk group for the onset of malignant cutaneous melanoma is exposure to ultraviolet (UV) radiation.^3^ Elderly people with a history of long working hours in the open air, such as fishermen and rural workers, should receive special attention. Another factor that directly influences the increase in the incidence of malignant neoplasms in elderly people is the population migration of retirees to coastal cities, resulting in a routine with a greater tendency to chronic photoexposure and the development of dermatoses.^5^

Although many studies demonstrate the behavior of mortality rates related to malignant melanoma of the skin worldwide, little is known about the temporal trend of mortality from this disease in Brazil in recent years, especially in the elderly population.^1^, ^9^, ^10^, ^11^ Studies are needed to indicate how the particularities related to old age influence the dynamics of this rate in each macro-region of the country. More information on this topic is needed in order to assist the development of public policies aimed at reducing the impact of this disease on the elderly. Thus, this article aims to analyze the temporal trend of mortality from malignant skin melanoma in elderly people in Brazil, between the years 2001 and 2016.

## Methods

This was a descriptive study of temporal trend of the mortality rate of malignant skin melanoma in the elderly, according to macro-regions and sex, from 2001 to 2016.

Data on melanoma deaths were extracted from the Mortality Information System (Sistema de Informações sobre Mortalidade [SIM]), and information on the resident population of Brazil and its macro-regions was obtained from the 2010 population census and population estimates from the Brazilian Institute of Geography and Statistics (Instituto Brasileiro de Geografia e Estatística [IBGE]) for the remaining years. The study included deaths from malignant skin melanoma (C43) of the International Classification of Diseases (ICD)-10. Deaths were studied according to the sex variable. Only individuals older than 60 years were included in the study. According the World Health Organization (WHO), the cut-off point for the beginning of old age in developing countries such as Brazil is the age of 60 years and, for developed nations, 65 years.^12^ Data referring to *in situ* melanoma (ICD−10: D.03) were not used.

For the description and analysis of the data, the gross mean coefficients of deaths due to malignant skin melanoma per macro-region were calculated for each year in each sex. The coefficients were calculated for every 100,000 inhabitants.

A trend analysis of mortality rates was performed for Brazil and its macro-regions, using the simple linear regression statistical technique, performed with Stata software, v. 14.0 (StataCorp, Texas, United States).

Mortality rates were considered as the response variable, also called the dependent variable, and the explanatory variable, or independent variable, was the years of the study. A statistically significant linear trend was admitted only when its probability of having occurred was equal to or less than 0.05, *i.e*., p ≤ 5%.

As this is a study in which public domain data were used without identifying individuals, the need for Research Ethics Committee approval was waived.

## Results

Between 2001 and 2016, 2,712 deaths from malignant skin melanoma in the elderly were registered in Brazil. The majority (56.8%) occurred in the male population. A constant increase in the number of deaths over the years has been observed.

The mortality coefficient in the general population (both sexes) of elderly people due to malignant skin melanoma rose from 3.83 per 100,000 inhabitants in 2001 to 4.47 per 100,000 inhabitants in 2016, representing an increase of approximately 16.7%. In the different macro-regions, with the exception of the Southeastern region, growth was also observed in both sexes, when comparing the years 2001 and 2016. A reduction in mortality rates for females in the Northern region (approximately 6%) was observed (Table 1).

**Table 1:** Mortality coefficient for elderly patients due to malignant skin melanoma, according to macro-regions and sex. Brazil, 2001 to 2016.

Region	2001	2004	2007	2010	2013	2016	2001/2016 (%)^a^
North							
Male	1.13	2.07	3.52	1.88	2.01	3.02	+167.3%
Female	1.13	1.02	1.36	1.00	1.40	1.06	-6.2%
Both	1.13	1.54	2.42	1.43	1.69	2.01	+77.9
Northeast							
Male	1.45	1.33	3.10	3.59	3.88	3.38	+133.1
Female	1.23	0.85	1.64	1.54	1.56	2.07	+68.3
Both	1.33	1.06	2.28	2.43	2.57	2.64	+98.5
Southeast							
Male	6.14	4.84	5.61	6.00	5.84	5.89	-4.1
Female	3.72	3.36	3.30	3.93	3.38	3.27	-12.1
Both	4.77	4.00	4.30	4.83	4.45	4.42	-7.3
South							
Male	9.20	9.78	9.22	8.69	11.04	10.55	+14.7
Female	4.42	5.05	7.34	6.79	5.84	6.01	+36.0
Both	6.54	7.15	8.17	7.63	8.15	8.04	+22.9
Midwest							
Male	3.36	2.78	4.30	4.48	3.88	4.99	+48.5
Female	2.49	1.31	2.64	2.27	2.35	3.76	+51.0
Both	2.92	2.02	3.44	3.48	3.07	4.33	+48.3
Brazil							
Male	4.89	4.42	5.35	5.54	5.89	5.86	+19.8
Female	2.98	2.75	3.40	3.56	3.18	3.36	+12.8
Both	3.83	3.49	4.26	4.43	4.38	4.47	+16.7

a Percentage increase (+) or decrease (-), comparing the years 2001 and 2016. (Rates calculated for every 100,000 inhabitants).

**Figure 1:**
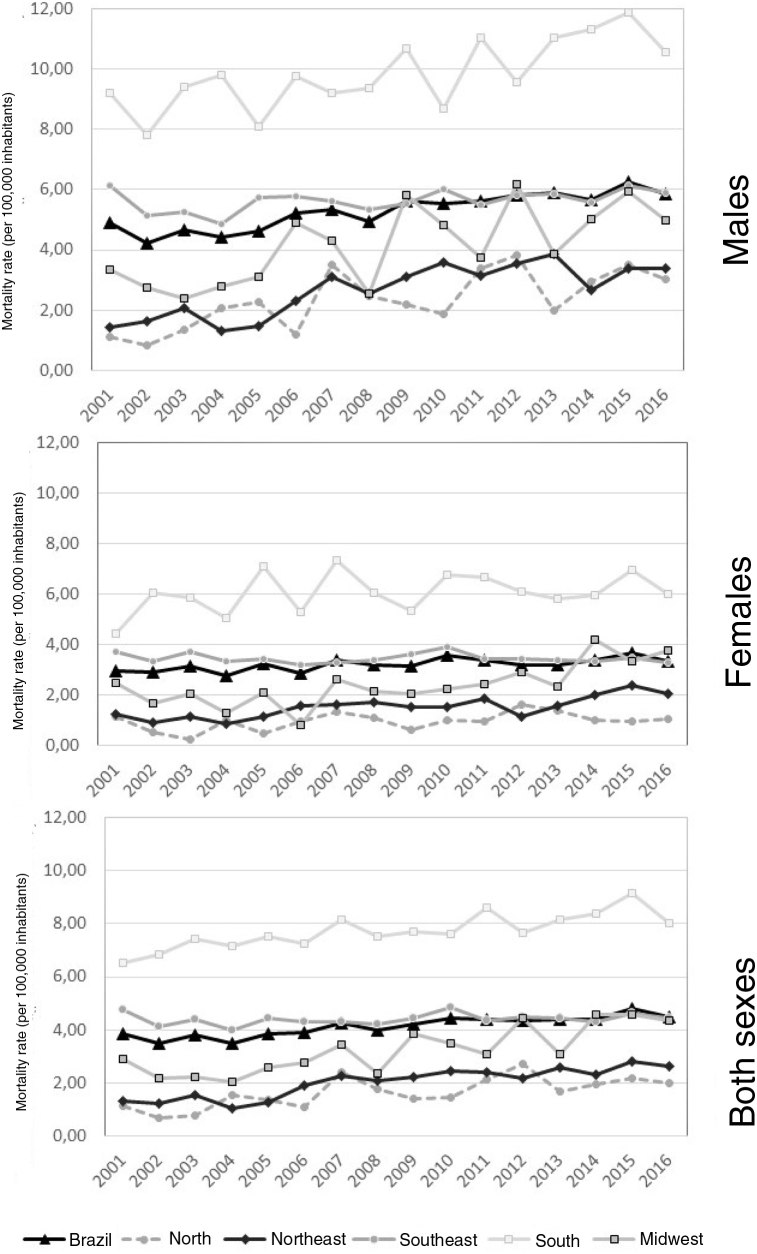
Trend in mortality rates due to malignant skin melanoma in the elderly, per 100,000 inhabitants, in males, females and both sexes. Brazil, 2001 to 2016.

Fig. 1 shows that the Southern region of Brazil had the highest mortality rates in all years of the analysis, ranging from 6.54 in 2001 to 8.04 in 2016 (both sexes).

In females, a tendency of increase in mortality rates due to malignant skin melanoma was observed in the Northeast (p ≤ 0.001), Midwest (p = 0.002), and Brazil as a whole (p = 0.003). In males, an upward trend was observed in all regions, except for the Southeastern region. Moreover, the largest increase was observed in the Midwestern region, with 0.18 deaths per 100,000 men. For both sexes, there was also an upward trend in all regions, with the exception of the Southeastern region (Table 2).

**Table 2:** Annual trend in mortality rates from malignant skin melanoma in the elderly, according to sex and macro-regions in Brazil, 2001 to 2016.

Macro-regions	Mean annual variation (95% CI)^a^	Interpretation	p-value	R² b
North				
Male	0.14 (0.06 − 0.22)	Increase	0.002	0.48
Female	0.03 (-0.00 − 0.07)	Oscillation	0.086	0.13
Both	0.08 (0.03 − 0.13)	Increase	0.002	0.46
Northeast				
Male	0.14 (0.09 − 0.20)	Increase	< 0.001	0.66
Female	0.07 (0.04 − 0.10)	Increase	< 0.001	0.59
Both	0.10 (0.07 − 0.13)	Increase	< 0.001	0.79
Southeast				
Male	0.03 (-0.00 − 0.07)	Oscillation	0.087	0.13
Female	-0.00 (-0.03 − 0.01)	Oscillation	0.482	-0.03
Both	0.01 (-0.01 − 0.03)	Oscillation	0.379	-0.01
South				
Male	0.17 (0.08 − 0.27)	Increase	0.001	0.51
Female	0.05 (-0.02 − 0.14)	Oscillation	0.171	0.06
Both	0.11 (0.06 − 0.15)	Increase	< 0.001	0.64
Midwest				
Male	0.18 (0.07 − 0.29)	Increase	0.003	0.44
Female	0.12 (0.05 − 0.20)	Increase	0.002	0.46
Both	0.15 (0.08 − 0.21)	Increase	< 0.001	0.63
Brazil				
Male	0.11 (0.08 − 0.14)	Increase	< 0.001	0.80
Female	0.03 (0.01 − 0.05)	Increase	0.003	0.45
Both	0.07 (0.05 − 0.09)	Increase	< 0.001	0.78

a 95% confidence interval.

b Adjusted R^2^.

## Discussion

The increase in mortality among elderly people due to malignant skin melanoma in Brazil and in its different macro-regions follows the trend observed worldwide in recent decades.^1^ The absolute numbers of deaths from this disease in the country have also grown over the years, following the gradual growth of the elderly Brazilian population. This increase is particularly evident in the Northeast, Midwest, and Brazil as a whole, where there was a significant increase for both sexes. In addition, during the analyses, higher coefficients were observed for the elderly male population when compared with those of the elderly female population.

The increase in mortality indicates a potential public health challenge for the coming decades. Continuous surveillance in this age group is necessary, due to the peculiarities of the disease at older ages.^4^ When compared with younger groups, melanoma mortality is significantly higher in the elderly, establishing age as a poor prognostic factor. In the elderly, the incidence of thicker lesions with a higher mitotic index and ulcerations is high, representing cancer with worse evolution.^4^, ^5^ Furthermore, it is highlighted that the elderly population has less capacity to repair nuclear deoxyribonucleic acid (DNA). Intense sun exposure has a cumulative effect, indicating greater chances of the appearance of malignant neoplasms.^13^

In the elderly population, the disease is also frequently associated with other pre-existing comorbidities, directly affecting the prognosis of these patients. Other possible explanations for the late discovery of malignant skin diseases in the elderly are the greater difficulty in detecting lesions in less evident areas such as the scalp and back, a higher prevalence of vision defects, less importance given to aesthetic aspects and self-care of the skin, and confusion with natural and benign skin changes during senescence.^4^, ^5^ Moreover, preventive measures such as campaigns to encourage photoprotection, campaigns to advise against the use of artificial tanning booths, and screening for these malignant neoplasms are normally aimed at younger age groups.^14^

In the Southern and Northern macroregions, the trend of increasing mortality rates in females did not follow that of males, indicating a fluctuation in the period. In addition, higher coefficients were observed for the elderly male population. It is suggested that women are diagnosed at earlier stages of the disease, mainly due to the greater use of health services, increasing the chances of cure and avoiding worse health outcomes. This behavior, in general, is attributed to greater self-perception of signs and symptoms, greater health concerns, and greater adherence to preventive campaigns.^15^ Franzon et al., when analyzing the medical records of patients diagnosed with melanoma for five years in a service specialized in skin cancer in the city of Curitiba/PR, identified that men had a later diagnosis of melanoma in relation to women.^16^

In the Southeastern macro-region, which has the highest gross domestic product (GDP) per capita according to data from the Institute of Research and Applied Economics (Instituto de Pesquisa e Economia Aplicada [IPEA]), an oscillation in the mortality coefficients between the years 2001 and 2016 was observed.^17^ These data corroborate those of studies that indicate a tendency towards stabilization in the number of deaths from the disease in regions with greater socioeconomic development.^1^ This behavior is observed mainly in the United States, Australia, and some Nordic countries.^9^, ^18^, ^19^ These studies indicate that, over the last decades, in these regions, the mortality rates from malignant melanoma have grown at a significantly lower rate in relation to the rate of growth of the disease incidence rates. This suggests that thinner, less aggressive lesions with better prognosis are possibly being diagnosed.

The impact of factors such as the historical construction of populations should also be emphasized. The Southern region of Brazil had the highest mortality rates in all years of the analysis, even though it is a region of high latitude and temperate climate. The strong colonization of these regions by European descendants with light skin can influence the behavior of the disease, since one of the risk factors for the development of this neoplasm is skin color.^3^ In a study by Gandini et al., it was concluded that the relative risk of developing melanoma was 2.06 for individuals with fair skin when compared to individuals with dark skin.^20^ In addition, these data also corroborate that of the study by Mendes et al., who assessed the mortality attributed to melanoma in Brazil during the period 1980 − 2005 for the general population and found an increase in mortality rates in the Southern region, highlighting the predictability of this scenario.^21^

In the Northern region, an increasing trend was observed for men, women, and both sexes combined. This may be linked to the high incidence of ultraviolet rays in this region, one of the main risk factors for the onset of malignant melanoma. Data from the National Institute for Space Research (Instituto Nacional de Pesquisas Espaciais [INPE]) show that the UV index of some Brazilian capitals is at levels considered to be very high or extreme, with the majority of these capitals being located in the Northeast of Brazil.^22^ However, an improvement in the quality of information cannot be ruled out as the cause for the increase in mortality in this Brazilian region.

As a possible limitation of the present study, secondary databases are directly influenced by the quality of death certificate completion and their heterogeneous scope in Brazilian regions. Even a macro-regional analysis can suffer numerous variances within its territory. It is necessary that new studies are carried out to allow other associations to better understand the mortality linked to malignant melanoma, evaluating other parameters, such as the area of residence (urban, rural), anatomical location, income, and/or educational status. However, SIM is a nationwide system and represents an important source of registration of deaths in the country, so its improvement and strengthening should be a priority for the management of information on mortality.

## Conclusions

There was a tendency towards increased mortality in the elderly population, in both sexes, for all regions of Brazil (except for the Southeastern region). Thus, it is highlighted that, in addition to early detection and diagnosis, there is a need for advances in the ways of distinguishing between aggressive and indolent melanomas, in order to target more effective treatment strategies for skin cancer. Prevention through campaigns and the population's awareness of this type of neoplasia should be a priority in public healthcare in Brazil, with special attention to population groups at risk, such as the elderly. In this subject, it is essential that access to therapeutic modalities at the different levels of complexity of the healthcare system is also guaranteed (regular clinical follow-up, availability of complementary exams, consultations with specialists, surgical procedures, and medications).

## Financial support

None declared.

## Authors’ contributions

Rodrigo Vasconi Sáez Brown: Statistical analysis; approval of the final version of the manuscript; conception and planning of the study; elaboration and writing of the manuscript; obtaining, analyzing, and interpreting the data; effective participation in research guidance; intellectual participation in propaedeutic conduct and/or therapeutics of studied cases; critical literature review; critical review of the manuscript.

Danúbia Hillesheim: Statistical analysis; approval of the final version of the manuscript; conception and planning of the study; elaboration and writing of the manuscript; obtaining, analyzing, and interpreting the data; effective participation in research orientation; intellectual participation in propaedeutic and/or therapeutic conduct of studied cases; critical review of the literature; critical review of the manuscript.

Yaná Tamara Tomasi: Statistical analysis; approval of the final version of the manuscript; conception and planning of the study; elaboration and writing of the manuscript; obtaining, analyzing, and interpreting the data; effective participation in research orientation; intellectual participation in propaedeutic and/or therapeutic conduct of studied cases; critical review of the literature; critical review of the manuscript.

Daniel Holthausen Nunes: Statistical analysis; approval of the final version of the manuscript; conception and planning of the study; elaboration and writing of the manuscript; obtaining, analyzing, and interpreting the data; effective participation in research orientation; intellectual participation in propaedeutic and/or therapeutic conduct of studied cases; critical review of the literature; critical review of the manuscript.

## Conflicts of interest

None declared.
